# Maternal glucose intolerance during pregnancy affects offspring POMC expression and results in adult metabolic alterations in a sex-dependent manner

**DOI:** 10.3389/fendo.2023.1189207

**Published:** 2023-06-15

**Authors:** Marina Galleazzo Martins, Zachary Silver, Kiara Ayoub, Lindsay Hyland, Barbara Woodside, Ana Carolina Inhasz Kiss, Alfonso Abizaid

**Affiliations:** ^1^ Department of Neuroscience, Carleton University, Ottawa, ON, Canada; ^2^ Department of Physiology, Institute of Biosciences of the University of São Paulo (IB/USP), São Paulo, São Paulo, Brazil; ^3^ Department of Structural and Functional Biology, Institute of Biosciences, São Paulo State University (Unesp), Botucatu, São Paulo, Brazil

**Keywords:** pregnancy, streptozotocin, glucose tolerance, metabolic programming, hypothalamus, POMC, gestational diabetes

## Abstract

**Introduction:**

Gestational diabetes (GDM) is associated with negative outcomes in mothers and their offspring, including greater risks of macrosomia at birth and the development of metabolic disorders. While these outcomes are well-established, the mechanisms by which this increased metabolic vulnerability is conferred on the offspring are comparatively lacking. One proposed mechanism is that maternal glycemic dysregulation alters the development of the hypothalamic regions related to metabolism and energy balance.

**Methods:**

To investigate this possibility, in this study, we first examined the effects of STZ-induced maternal glucose intolerance on the offspring on pregnancy day (PD) 19, and, in a second experiment, in early adulthood (postnatal day (PND) 60). Whether effects would be influenced by sex, or exposure of offspring to a high-fat diet was also investigated. The impact of maternal STZ treatment on POMC neuron number in the ARC of offspring at both time points was also examined.

**Results:**

As expected, STZ administration on PD 7 decreased maternal glucose tolerance, and increased risk for macrosomia, and loss of pups at birth. Offspring of STZ-treated mothers were also more vulnerable to developing metabolic impairments in adulthood. These were accompanied by sex-specific effects of maternal STZ treatment in the offspring, including fewer POMC neurons in the ARC of female but not male infants in late pregnancy and a higher number of POMC neurons in the ARC of both male and female adult offspring of STZ-treated dams, which was exacerbated in females exposed to a high-fat diet after weaning.

**Discussion:**

This work suggests that maternal hyperglycemia induced by STZ treatment, in combination with early-life exposure to an obesogenic diet, leads to adult metabolic alterations that correlate with the increased hypothalamic expression of POMC, showing that maternal glycemic dysregulation can impact the development of hypothalamic circuits regulating energy state with a stronger impact on female offspring.

## 1 Introduction

Gestational diabetes (GDM) affects 5-10% of all pregnancies worldwide and is a metabolic disorder mostly characterized by hyperglycemia, in which women without a prior diagnosis of diabetes develop abnormally high blood glucose levels during pregnancy (1) as well as an exacerbation of the typical state of insulin resistance that occurs towards the second half of pregnancy and critical for fetal growth and development (2, 3). If not properly diagnosed and treated, poor hyperglycemia management in women with GDM can result in negative consequences both for the mother and their offspring. These include a greater risk of obstetric complications, such as preterm birth, preeclampsia, and c-section (2, 4–6). In addition, children born to women with GDM are at a greater risk of being classified as macrosomic at birth (2, 4, 5, 7–9), usually showing increased body fat independently of macrosomia (7, 10) both of which are major predictors of long-term health (11, 12). Some effects of GDM on the offspring may be sex-dependent. For example, in humans, male offspring of GDM mothers are at a higher risk of congenital malformations and respiratory disorders when compared to their female counterparts (13).

The effects of GDM on the offspring persist across the lifespan. Children born to mothers diagnosed with GDM have a higher risk of developing obesity, type II diabetes, and metabolic syndrome (3, 14–18). Moreover, women whose mothers were diagnosed with GDM were more likely to develop it during their pregnancies (18). While there is considerable evidence supporting the links between GDM and greater vulnerability to the development of metabolic disorders in the offspring in adulthood, little is known about the mechanisms underlying these links. Some studies do suggest that maternal hyperglycemia alters the development of brain regions related to the regulation of metabolism and energy balance in their offspring. For example, children exposed to GDM show elevated hypothalamic activation in response to oral glucose, and greater activation in the orbitofrontal cortex in response to food cues (19), suggesting that GDM affects brain circuitry related to energy balance and food intake control.

One way to understand how GDM impacts the development of brain regions associated with the regulation of energy balance is to generate pre-clinical animal models that mimic aspects of this altered metabolic state in pregnant laboratory animals and look at the impact of these manipulations on brain systems known to regulate metabolic function. Several preclinical models have emerged to study GDM, including models of diet-induced maternal hyperglycemia, and models of hypoinsulinemia produced by agents that deplete the pancreas of insulin secreting ß-cells, using alloxan or streptozotocin (STZ). Experimentally, the administration of STZ has been widely used to develop hyperglycemia and decreased glucose clearance in rodents (20). In most rodent studies, however, STZ administration leads to severe hyperglycemia, with fasting glycemia over 300 mg/dL, which is rarely observed in women with GDM. Alternative models of mild hyperglycemia have been developed using lower doses of STZ, leading to maternal glucose levels between 120 and 300 mg/dL which are more similar to those seen in women with GDM (21, 22). This is noteworthy because poor fetal outcomes have been described even with mildly increased glucose levels (23). For example, there is a positive correlation between maternal glycemia and offspring body fat and hyperglycemia during childhood, even in mothers that do not meet the clinical criteria for GDM (24–26).

Using STZ administration in early pregnancy in rats to induce maternal hyperglycemia, Plagemann et al. observed a reduction in the size of the paraventricular (PVN), ventromedial (VMH), and arcuate (ARC) nuclei of the hypothalamus in 21-day-old offspring (27). A similar study in pregnant mice found a reduction in the projections from AgRP/NPY and POMC neurons of the ARC to the PVN in the adult offspring from STZ-treated mothers compared to controls, which occurred despite an increased number of POMC neurons in the ARC (28). Furthermore, these offspring also showed decreased leptin sensitivity as reflected in decreased leptin-induced STAT3 activation in POMC neurons (28), a crucial step in leptin’s effects on energy balance. Overall, these data strongly suggest that maternal hyperglycemia has significant, long-lasting effects on hypothalamic anatomy and function in the offspring and it is reasonable to speculate that these changes may be implicated in the development of various metabolic disorders in offspring following GDM. However, these studies focused on outcomes in male offspring, and it is not known whether similar effects occur in females.

In the current study, we address this gap by examining the effects of STZ-induced maternal glucose intolerance on offspring outcomes at the end of pregnancy and as these offspring reach adulthood, adding sex as a variable to explore the potential sex-dependent effects of maternal hyperglycemia on offspring development. Furthermore, we examined whether maternal STZ-induced glucose intolerance increases offspring vulnerability to the development of metabolic disorders later in life when challenged with a high-fat diet.

## 2 Materials and methods

### 2.1 Animals

Male and female 8-week-old Wistar rats were obtained from Charles River (St. Constant, Québec). Upon arrival in our facility, male rats were housed individually, and female rats were housed in pairs in clear plexiglass cages with nesting material provided as enrichment and kept under controlled temperature (20°C) and humidity (40%), on a 12/12 h light/dark cycle. Rats were allowed to acclimate to our facility under these conditions for a period of 10-14 days. All rats had free access to chow (3.1 kcal/g, 2018 Teklad Global) and tap water. All experimental procedures were approved by the Carleton University Animal Care Committee and followed the guidelines established by the Canadian Council on Animal Care (CCAC).

### 2.2 Gestational diabetes model

All animals used in this project were procured at the same time but assigned to two separate experiments, one examining the offspring outcomes on pregnancy day (PD) 19 and the second examining effects in adulthood.

#### 2.2.1 Mating and STZ administration

Following acclimation, female rats were housed with males to induce mating. For the ensuing days, vaginal smears were collected daily in the morning from females until spermatozoa were found in the sample. This was considered PD 0. Dams were then individually housed and received tap water and chow ad libitum. Maternal body weight and food intake were measured daily during pregnancy and, where appropriate across lactation. These data were used to calculate maternal weight gain and caloric intake. On PD 7, female rats were randomly assigned to two experimental groups: STZ (n = 15), in which they received 35 mg/kg of streptozotocin (STZ, SIGMA Chemical Company, St. Louis, Millstone) intraperitoneally, diluted in citrate buffer (0,1 M, pH 4,5); or Control (n = 9), in which they received citrate buffer intraperitoneally [adapted from (29)].

#### 2.2.2 Oral glucose tolerance test

On PD 16, all dams were given an oral glucose tolerance test (OGTT). After fasting for 6 h, dams received a 2 g/kg glucose solution (200 g/L) by gavage. Blood samples were obtained from a tail snip 0, 15, 30, 60, and 120 min after glucose administration, and blood glucose was measured with a glucometer (Contour Glucose Meter, Ascensia Diabetes Care Canada). Blood glucose changes across the glycemic curve were assessed by calculating the total area under the curve (AUC) with the trapezoid method (30).

### 2.3 Experiment I: Maternal and fetal outcomes on late pregnancy

To evaluate the effects of maternal hyperglycemia on fetal outcome during development and its impact on the neural substrates that control food intake, a subset of Control (n = 4) and STZ-treated pregnant females (n = 7) were rapidly euthanized on PD 19 by CO2 inhalation and decapitated to collect maternal trunk blood for hormonal assays. Within two minutes of decapitation, the uterine horns were exposed through a mid-line incision, and fetuses and their respective placentae were dissected and weighed. Placental efficiency was calculated as fetal body weight/placental weight. Half of the fetal brains and placentae were fixed in freshly made 4% paraformaldehyde solution, pH 7.4, and then cryoprotected in a 30% sucrose solution, while the other half were quickly frozen in dry ice and stored at -80°C. Fetal sex was confirmed through genotyping placental samples by an outsourced commercial genotyping services provider using the SRY gene to detect males from females (Transnetyx Inc. Cordova, TN, USA).

#### 2.3.1 Maternal hormonal levels

Maternal blood samples were centrifuged at 5000 RPM for 15 minutes, at 4°C, and serum was separated for future analysis. Insulin and leptin levels were assessed with commercial immunoassay kits (Rat Leptin Elisa, Millipore, #EZRL-83K; Rat/Mouse Insulin ELISA, Millipore, #EZRMI-13K), following the manufacturer’s instructions. Samples were evaluated in duplicate and the intra-assay coefficients of variability were 14.3% and 11.8% for the leptin and insulin immunoassays, respectively. Triglyceride levels were also analyzed through a commercial colorimetric assay kit following the manufacturer’s instructions (Cayman Chemicals, #10010303).

#### 2.3.2 Immunohistochemistry for proopiomelanocortin peptide in PD 19 brains

Immunohistochemical analyses were performed in male and female fetal offspring brains (PD 19) drop-fixed on 4% PFA. After 72 h in 4% PFA, brains were cryoprotected in a 30% sucrose solution for a minimum of 72 h. Heads were embedded in cryomatrix and mounted frozen on a cutting chuck in a cryostat (Thermo Fisher) with the anterior portion of the head facing the blade. Full heads were serially sliced at -22°C and 40 μm slices were mounted directly onto gelatin-coated slides and stored at -20°C until processed for immunohistochemical staining.

Immunohistochemical staining for proopiomelanocortin (POMC) in the ARC took place at room temperature (~21°C). Mounted tissue sections were incubated with 1:20,000 POMC primary antibody (anti-POMC 27-52, Phoenix Pharmaceuticals Inc., #H-029-30) for 24h, followed by incubation with 1:250 biotinylated donkey-anti-rabbit (Jackson ImmunoResearch, #711-065-152) for 1 h. Avidin-biotin complexes were then formed using Vectastain^®^ Elite ABC-HRP kit (Vector Laboratories). Sections were then treated with 0.1% diaminobenzidine (DAB) and 10 μl of 1% hydrogen peroxide. Sections were hand-shaken in the DAB-hydrogen peroxide solution for 3 min before being washed with 1x PBS. Sections were then imaged on a Zeiss Axioplan microscope at 20x magnification to collect pictures of all sections from the arcuate nucleus with POMC-positve neurons following Paxinos et al. (31). Fetal POMC neuron number in the ARC was then quantified as the average number of POMC-positive cells per ARC section using ImageJ by a researcher blind to animals’ experimental group.

### 2.4 Experiment II: Offspring outcomes in adulthood

To evaluate offspring outcomes in adulthood and the susceptibility of offspring of hyperglycemic dams to the development of obesity and diabetes following high-fat diet exposure, a subset of Control (n = 7) and STZ treated pregnant females (n = 7) were allowed to give birth naturally. During pregnancy and lactation, dams had access to standard chow (3.1 kcal/g, 2018 Teklad Global), and their body weight and food intake were measured daily. Body weight gain during pregnancy and maternal weight change across lactation, as well as overall caloric intake, were calculated. On postnatal day (PND) 1, litters were examined to determine litter size, sex ratios, and average pup size, and then these were culled so that each dam nursed four male and four female pups. Litters remained with their dams until weaning at PND 21. At weaning, two males and two females from each litter were assigned to the Chow condition, males (n = 14) and females (n = 14), and given access to standard chow (2.9 kcal/g, 2014 Teklad Global) from weaning to PND 60. The remaining two males and females from each litter were assigned to the high-fat diet (HFD) condition, males (n = 14) and females (n = 14), and given access only to a high-fat diet (5.21 kcal/g, 60% kcal from fat, D12492 Research Diets Inc.) from weaning to PND 60. Within diet conditions, same sex littermates were pair housed throughout the experiment. Body weight and food intake were measured weekly and the average body weight for each littermate pair was used in the analysis, thus the litter was considered the experimental unit throughout.

#### 2.4.1 Intraperitoneal glucose tolerance test

After PND 60, all rats were given an intraperitoneal glucose tolerance test (IpGTT). After a 6 h fast, rats received a 2 g/kg glucose solution (200 g/L) *via* intraperitoneal injection. As previously described (section 2.2.2), blood samples were obtained from a tail snip 0, 15, 30, 60, and 120 min, and blood glucose was measured with a glucometer (Contour Glucose Meter, Ascensia Diabetes Care Canada). Changes across the glycemic curve were assessed by calculating the total area under the curve (AUC) with the trapezoid method (30).

The following day, half of the rats in each group were euthanized by decapitation for fat deposition analyses, while the other half was deeply anesthetized and perfused with 0.9% saline followed by a freshly made 4% PFA solution, pH 7.4. Brains were then removed and post-fixed in 4% PFA for 48 h and then cryoprotected in a 30% sucrose solution for at least 48 h. Retroperitoneal and gonadal fat pads in male and female offspring that were decapitated were dissected and weighed. Adiposity was calculated as (absolute fat pad weight/body weight) x 100.

#### 2.4.2 POMC immunohistochemistry in adult brains

Immunohistochemical analysis was performed on 4% PFA-perfused brain tissue from adult offspring. Brain tissue was coronally sliced at 50 µm width on a cryostat at -20°C. Four sets of sections from each animal were placed in glycerol-based cryoprotectant and stored at -20°C until immunohistochemical staining. For POMC staining in the arcuate nucleus, one set of tissue sections was incubated with 1:20,000 POMC primary antibody (anti-POMC 27-52; Phoenix Pharmaceuticals Inc., #H-029-30) for 24 h, followed by incubation with 1:250 biotinylated donkey-anti-rabbit (Jackson ImmunoResearch., #711-065-152) for 1 h. Avidin-biotin complexes were then formed using Vectastain^®^ Elite ABC-HRP kit (Vector Laboratories). Sections were then treated with 1 mL of 0.1% diaminobenzidine (DAB) and 10 µL of 1% hydrogen peroxide. Sections were hand-shaken in the DAB-hydrogen peroxide solution for 3 min before being washed with 1x PBS and stored at 4°C before being slide-mounted. Sections were dehydrated through a series of EtOH dehydration and cleared using Clearene solvent (Leica Biosystems). Arcuate POMC expression was quantified as the average number of POMC-positive cells counted bilaterally across all ARC sections for an animal. Cell quantification was performed using ImageJ by a researcher blind to the animals’ experimental group. In addition, POMC projections were evaluated in the PVN as the total integrated optical density (IOD) of POMC-positive fibers bilaterally across all PVN sections. IOD analysis was performed using ImageJ and a greyscale filter (0.04-1.0, Edmund Optics, #32-599) as a reference for optical density.

### 2.5 Statistical analyses

Data are expressed as mean ± standard error mean. Data from maternal AUC, maternal body composition and caloric intake, litter size, offspring birth, placental weight, and maternal hormonal levels were analyzed with an independent t-test. Data from maternal glycemic curves were analyzed with repeated measures 2-way ANOVA, with time as the repeated measure and maternal metabolic state (normo- or hyperglycemic) as a between-subject factor. Data from offspring POMC neurons on PD 19 were analyzed with a 2-way ANOVA, with maternal metabolic state and sex as between-subject factors. Data from offspring body composition and total caloric intake, AUC, and offspring POMC neurons in adulthood were analyzed with a 3-way ANOVA, with maternal metabolic state, diet (standard chow or HFD), and sex as between-subject factors. Data from offspring’s glycemic curves were analyzed with repeated measures 4-way ANOVA, with time as the repeated measure, and maternal metabolic state, diet, and sex as between-subject factors. Significant interactions were investigated with simple main effects ANOVA or t-tests. F and p values and partial eta are reported for all ANOVA analyses. In all cases, statistical significance was set at alpha = .05. All statistical analyses were performed using SPSS (IBM, SPSS Statistics 22).

## 3 Results

### 3.1 Gestational diabetes model

**Figure 1:**
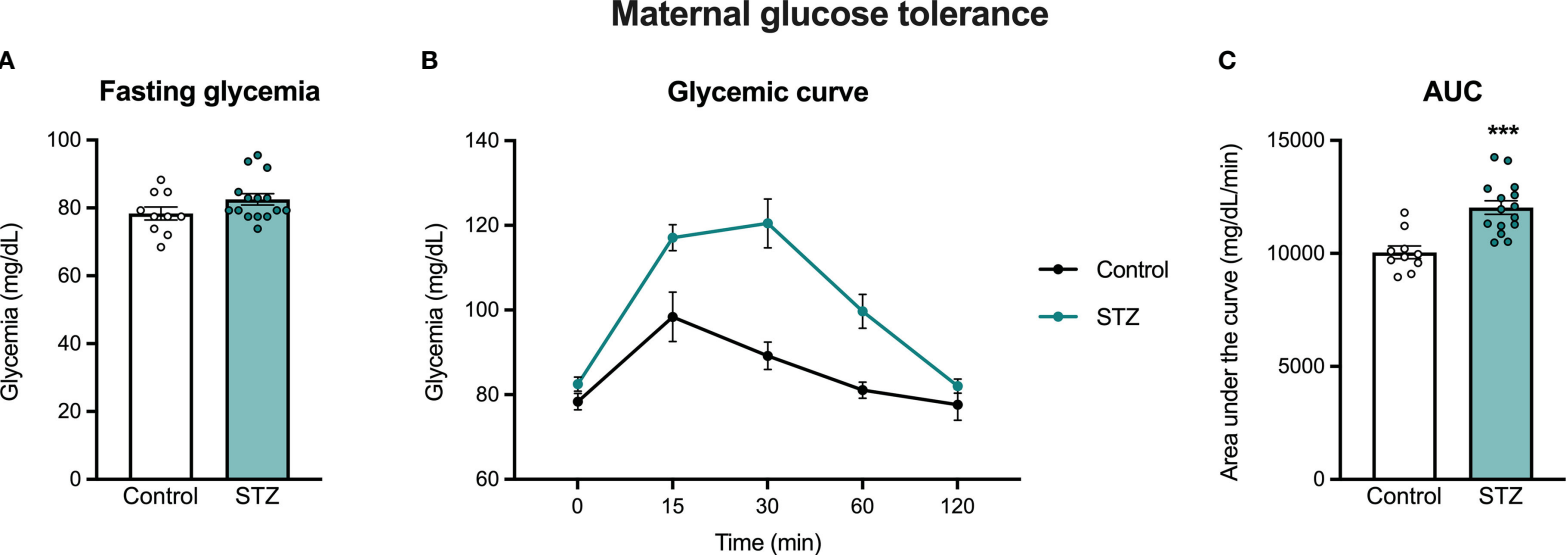
Maternal oral glucose tolerance on pregnancy day 16. STZ administration in early pregnancy did not increase fasting glycemia **(A)**, but increased glucose intolerance on PD 16 **(B, C)**. Values expressed as mean ± standard error of the mean (Control N = 10; STZ N = 15). ***p<0.001.

**Figure 1** shows the glycemic responses to the OGTT in control and STZ-treated pregnant rats. As shown in this figure, females treated with STZ during pregnancy showed glucose intolerance on PD 16, as reflected in a higher glycemic curve in the OGTT (**Figure 1B**; significant time x metabolism interaction, F (4,92)=6.193, p<.001, η=.212 and metabolism effect F (1,92)=21.33, p<.001, η=.481) and in the increased AUC (**Figure 1C**, t (23)=4.557, p<.001). Despite increased glucose intolerance, STZ-treated dams did not show impaired fasting glycemia (**Figure 1A**, p>.05).

### 3.2 Experiment I: Maternal and fetal outcomes on late pregnancy

#### 3.2.1 Maternal outcomes

Maternal STZ treatment did not change litter size, fetal body weight, or placental weight on litters harvested on PD 19, resulting in no differences in placental efficiency between groups (**Table 1**; p>.05).

**Table 1:** Maternal outcomes on PD 19.

	Control	STZ
Litter size	18.00 ± 1.23 (4)	17.71 ± 0.52 (7)
Fetal body weight (g)	2.04 ± 0.08 (4)	2.07 ± 0.05 (7)
Placental weight (g)	0.493 ± 0.03 (4)	0.520 ± 0.03 (7)
Placental efficiency	4.196 ± 0.16 (4)	4.082 ± 0.17 (7)
Insulin (ng/L)	16.02 ± 4.30 (5)	7.157 ± 2.26 (7)
Leptin (ng/L)	7.065 ± 2.14 (5)	3.444 ± 0.58* (7)
Triglycerides (mg/dL)	367.6 ± 69.68 (5)	300.7 ± 28.48 (7)

Values expressed as mean ± standard error of the mean. Numbers in parentheses indicate the N per group for each parameter. *p<0.05.

#### 3.2.2 Maternal hormonal levels

A one-tailed t-test showed a reduction in insulin levels on PD 19 in the STZ group when compared to the Control group (**Table 1**; t (10)=1.98, p<.05). Despite a decrease in leptin levels in the STZ group, that difference was not statically significant when compared to the Control group (**Table 1**; p>.05). Triglyceride levels also did not differ between STZ and Control dams (**Table 1**; p>.05).

#### 3.2.3 POMC expression in the ARC of PD 19 pups from STZ-treated or Control dams

**Figure 2:**
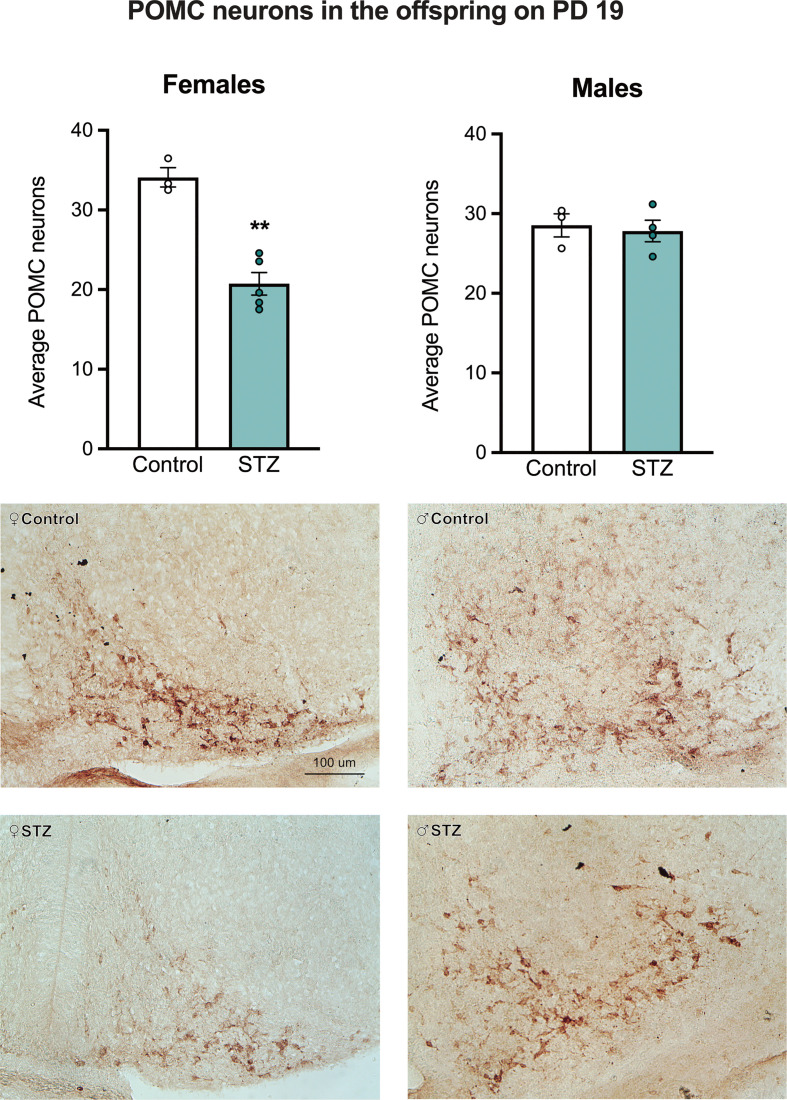
The average number of POMC neurons in the Arc of female and male offspring on PD 19. Maternal hyperglycemia reduced the number of POMC neurons in the female offspring, but not in males. Values expressed as mean ± standard error of the mean (Females: Control N = 3, STZ N = 5; Males: Control N = 3, STZ N = 4). The images represent POMC staining in the Arc in all experimental groups. The scale in the first image (100 µm) is valid for all the images. **p<0.01.

**Figure 2** shows sample images from the ARC of control and STZ treated PD 19 pups stained for POMC. As shown in this figure, maternal hyperglycemia reduced the number of POMC neurons in the ARC of female but not male offspring on PD 19, as determined by a metabolism x sex interaction effect followed by *post hoc* comparisons (see **Figure 2**; F (1,11)=18.867, p<.01, η=.632).

### 3.3 Experiment II: Offspring outcomes in adulthood

#### 3.3.1 Maternal weight change and food intake during pregnancy and lactation

Mild hyperglycemia did not result in changes in body weight gain, caloric intake, and fat deposition across either pregnancy or lactation (**Table 2**; p>.05).

**Table 2:** Maternal body composition and caloric intake during pregnancy and lactation.

	Control	STZ
Pregnancy		
Total body weight gain (g)	146.5 ± 12.22 (6)	140.7 ± 4.45 (15)
Total caloric intake (kcal)	1274 ± 96.49 (5)	1289 ± 31.42 (15)
Relative gonadal fat (%)	1.568 ± 0.03 (3)	1.102 ± 0.22 (7)
Relative retroperitoneal fat (%)	0.929 ± 0.22 (3)	0.808 ± 0.12 (7)
Lactation		
Total body weight change (g)	50.29 ± 2.71 (7)	58.14 ± 6.01 (7)
Total caloric intake (kcal)	2067 ± 80.49 (3)	2243 ± 51.42 (7)
Relative gonadal fat (%)	1.416 ± 0.17 (5)	1.075 ± 0.18 (7)
Relative retroperitoneal fat (%)	0.801 ± 0.15 (5)	0.948 ± 0.18 (7)

Values expressed as mean ± standard error of the mean. Numbers in parentheses indicate the N per group for each parameter.

#### 3.3.2 Pregnancy outcomes

**Figure 3:**
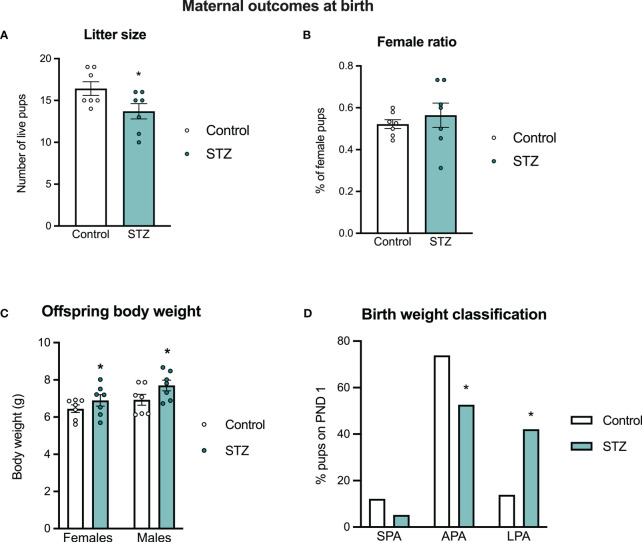
Maternal outcomes at birth. Maternal hyperglycemia reduced litter size at birth **(A)**, without changes in sex ratio **(B)**. Both female and male offspring born to hyperglycemic dams showed increased body weight at birth **(C)**, which was followed by a different distribution of pups classified asappropriate and large for pregnancy age in the hyperglycemic groups **(D)**. Values expressed as mean ± standard error of the mean (Control N = 7; STZ N = 7). *p<0.05.

Although we observed no differences in litter size between the control and STZ-treated dams in those killed on PD 19, there were differences when females gave birth naturally on PD 22. STZ-treated dams gave birth to smaller litters than controls (**Figure 3A**; t (12)=2.214, p<.05) and both male and female offspring born to STZ-treated dams were heavier at birth than those of control dams (**Figure 3C**; metabolism effect, F(1,24)=4.927, p<.05, η=.170). In hyperglycemic dams, there was a higher proportion of pups classified as large for pregnancy age at birth, followed by a reduction in the proportion of pups classified as appropriate (**Figure 3D**; Fisher’s test, p<.05). Sex ratio did not differ between litters of the two groups (**Figure 3B**; p>.05), however, male offspring were heavier than female offspring regardless of maternal metabolic state (sex effect, F(1,24)=5.388, p<.05, η=.183).

#### 3.3.3 Offspring body composition

**Figure 4:**
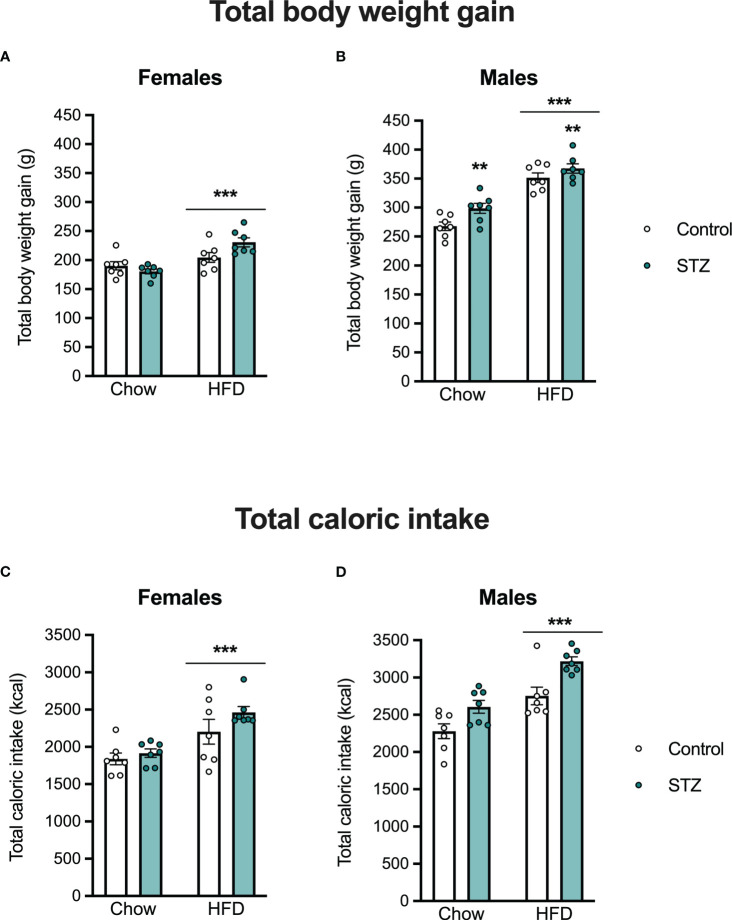
Female and male offspring body weight gain and caloric intake on PND 60. High-fat diet intake increased body weight gain and total caloric intake both in female **(A, C)** and male offspring **(B, D)**. Increased body weight gain was also seen in male offspring born to hyperglycemic dams. Values expressed as mean ± standard error of the mean (Control N = 7; STZ N = 7). **p<0.01; ***p<0.001.

**Figure 5:**
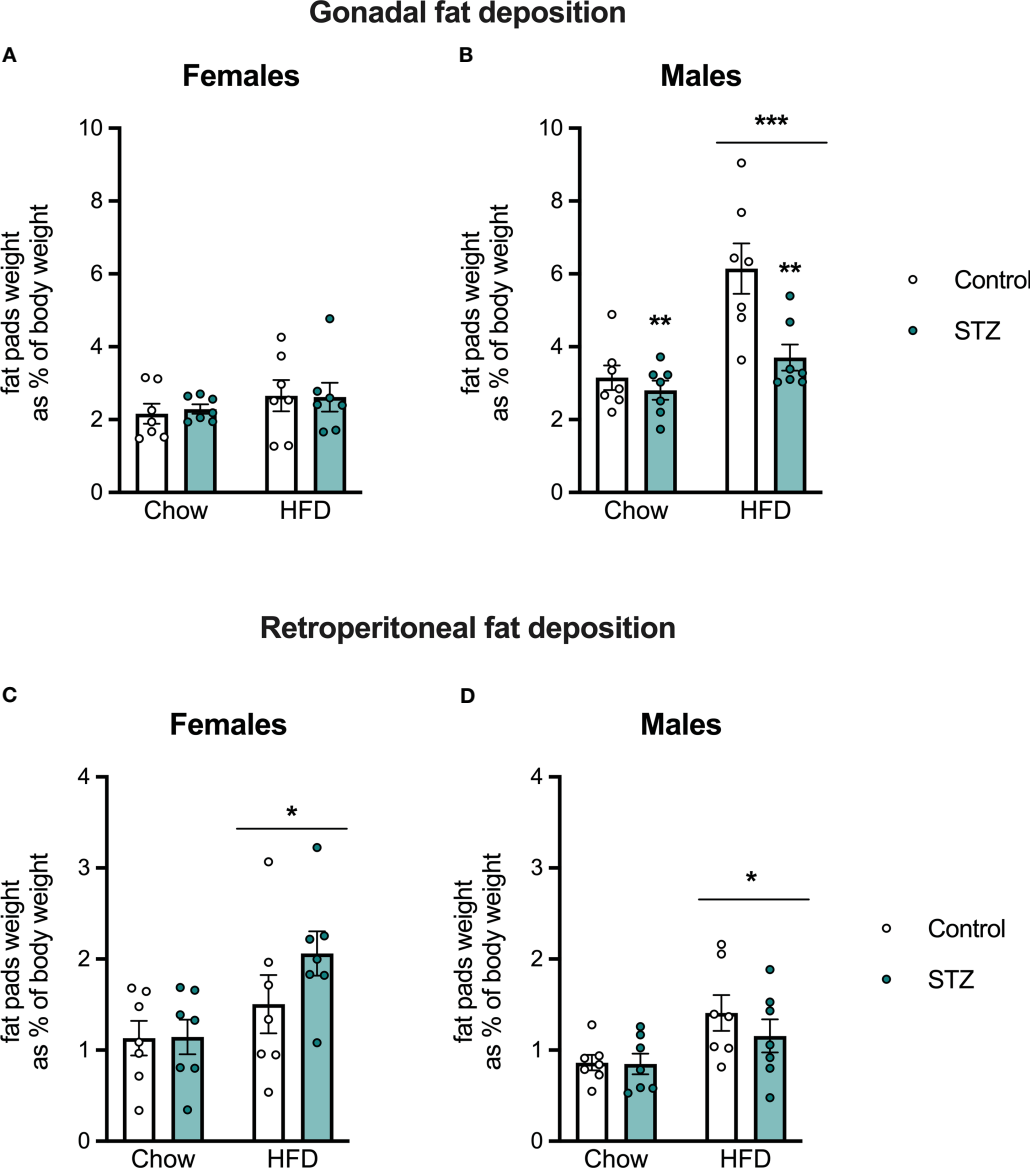
Female and male offspring fat deposition on PND 60. Despite no changes in female gonadal fat deposition **(A)**, high-fat diet intake increased gonadal fat in males **(B)** and retroperitoneal fat deposition both in females **(C)** and males **(D)**. Interestingly, maternal hyperglycemia decreased gonadal fat in males (B). Values expressed as mean ± standard error of the mean (Control N = 7; STZ N = 7). *p<0.05; **p<0.01; ***p<0.001.

Offspring body weight and food intake were measured weekly after weaning. As expected, there were sex differences in body weight gain (sex effect, F(1,48)=391.008, p<.001, η=.891) and caloric intake (sex effect, F(1,48)=49.315, p<.001, η=.507), so those outcomes were evaluated separately in male and female offspring (see **Figure 4**). In females, HFD intake led to an increased body weight gain (diet effect, F(1,24)=21.001, p<.001, η=.467), which was modified by the maternal hyperglycemia (metabolism x diet interaction, F(1,24)=6.414, p<.05, η=.211). Females born to hyperglycemic dams had a higher body weight gain following HFD intake (**Figure 4A**). Access to the HFD also increased total caloric intake in females (**Figure 4C**; diet effect, F(1,24)=35.706, p<.001, η=.598). This was accompanied by an increased retroperitoneal fat deposition (diet effect, F(1,24)=7.124, p<.05, η=.229), but not by differences in gonadal fat (**Figures 5A–C**; p>.05).

Exposure to an HFD intake also led to increased body weight gain (diet effect, F(1,24)=88.842, p<.001, η=.787) and total caloric intake (diet effect, F(1,24)=26.804, p<.001, η=.528) in the male offspring (**Figures 4B–D**). This increased body weight was associated with an increase in gonadal and retroperitoneal fat deposition after HFD (**Figure 5B**; diet effect, F(1,24)=18.180, p<.001, η=.444 and F(1,24)=7.892, p<.05, η=.247, respectively). Interestingly, maternal hyperglycemia increased body weight gain only in the male offspring (**Figure 4B**; metabolism effect, F(1,24)=8.314, p<.01, η=.257). However, maternal hyperglycemia and offspring HFD intake had opposing effects on gonadal fat deposition. Gonadal fat was reduced in males born to hyperglycemic dams (**Figure 5B**; metabolism effect, F(1,24)=9.859, p<.01, η=.291, and metabolism x diet interaction, F(1,24)=5.603, p<.05, η=.189).

#### 3.3.4 Offspring glucose tolerance

**Figure 6:**
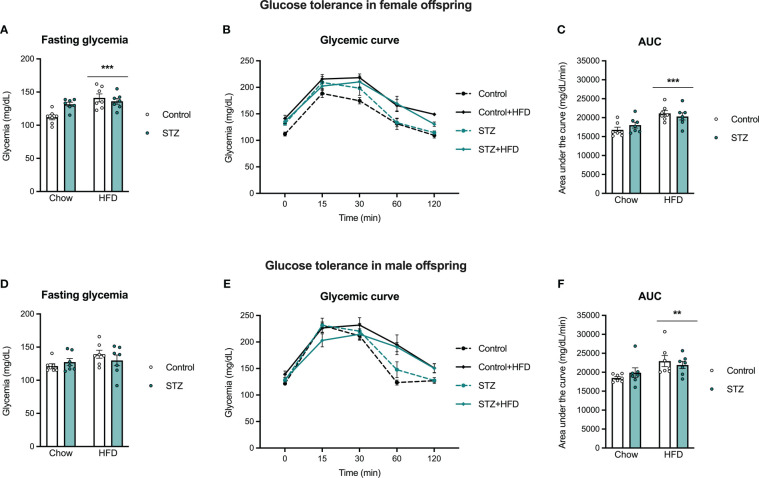
Female and male offspring glucose tolerance on PND 60. High-fat diet intake after weaning increased fasting glycemia in females **(A)**, but not in males **(D)**, which was significantly impaired by maternal hyperglycemia. Also, high-fat diet exposure increased glucose intolerance in female and male offspring, as seen in the glycemic curve **(B, E)** and the AUC **(C, F)**. Values expressed as mean ± standard error of the mean (Control N = 7; STZ N = 7). **p<0.01; ***p<0.001.

To evaluate the offspring’s vulnerability to developing glucose intolerance in adulthood, an intraperitoneal glucose tolerance test was performed on PND 60. Main effect analysis revealed sex differences in the offspring glycemic curve (sex effect, F(1,48)=6.939, p<.05, η=.126), and therefore glucose tolerance was evaluated in males and females separately (**Figure 6**). As expected, glucose administration increased glycemia across time in males and females (time effect, males: F(4,96)=187.1, p<.001, η=.886; females: F(4,96)=119.2, p<.001, η=.832). Also as expected, the HFD intake after weaning decreased glucose clearance in females (diet effect, F(1,24)=18.580, p<.001, η=.436), which was accompanied by an increased AUC (**Figures 6A, B**; diet effect, F(1,24)=17.474, p<.001, η=.421). In males, high-fat diet intake did not change glucose tolerance (p>.05), despite a significant time x diet interaction (F(4,96)=17.491, p<.001, η=.422), accompanied by an increased AUC (**Figures 6C, D**; diet effect, F(1,24)=8.759, p<.01, η=.267). Fasting glucose levels were also higher in females after a high-fat diet intake (diet effect, F(1,24)=14.609, p<.001, η=.378), but not in males (p>.05). In addition, there was a significant maternal metabolism x diet interaction on fasting glycemia in the female offspring (F(1,24)=7.727, p<.05, η=.244). High-fat diet intake increased glycemia in the females born to control dams (t(12)=-4.144, p<.001), but there were no differences in glycemic responses in offspring of STZ-treated dams regardless of the diet they were exposed to (**Figure 6A**; p>.05).

#### 3.3.5 Effects of maternal exposure to STZ on POMC neuron expression in the ARC of adult offspring and in response to a high-fat diet

**Figure 7:**
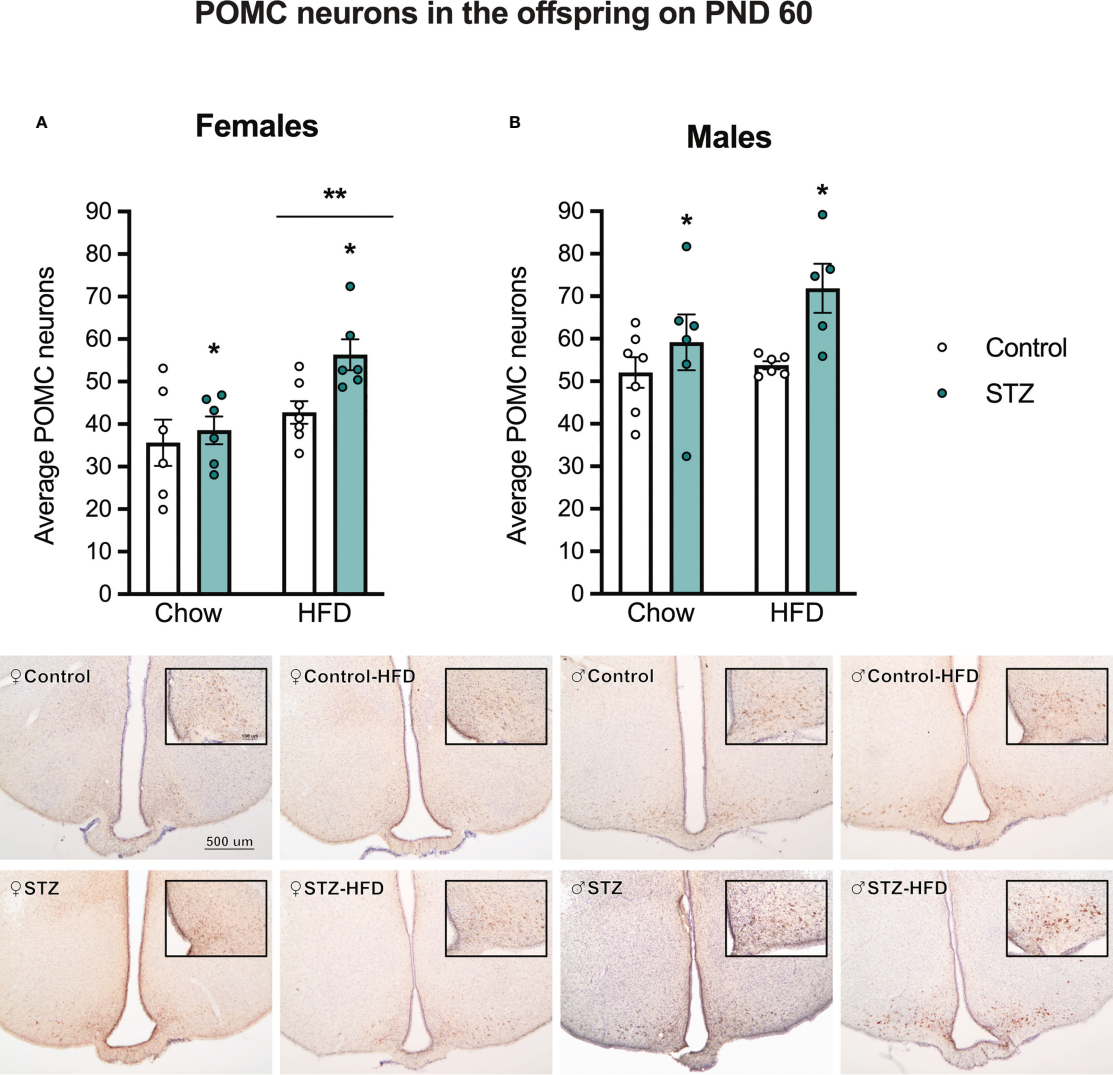
Average POMC neurons in female and male offspring on PND 60. Maternal hyperglycemia increased the number of POMC neurons in the Arc in both female **(A)** and male offspring **(B)**. Interestingly, a high-fat diet intake after weaning increased POMC neurons only in females. Values expressed as mean ± standard error of the mean (Females: Control-chow N = 6; Control-HFD N = 6; STZ-Chow N = 7; STZ-HFD N = 6. Males: Control-chow N = 7; Control-HFD N = 6; STZ-Chow N = 6; STZ-HFD N = 5). The images represent POMC staining in the Arc in all experimental groups. The scale in the first image and inset (500 and 100 µm respectively) are valid for all the images. *p<0.05; **p<0.01.

**Figure 7** shows sample images of the ARC from adult male and female offspring of STZ or vehicle-treated dams exposed to a standard or HFD. As shown in these images, the number of POMC neurons was significantly higher in males than in females (sex effect, F(1,41)=28.502, p<.001, η=.410). Furthermore, maternal STZ treatment resulted in a higher number of POMC neurons per section of the ARC of males (metabolism effect, F(1,20)=7.424, p<.05, η=.271) and females (metabolism effect, F(1,21)=4.695, p<.05, η=.183). Moreover, HFD intake after weaning increased the number of POMC neurons per section of the ARC only in female offspring (**Figure 7**; diet effect, F(1,21)=10.690, p<.01, η=.337) regardless of maternal treatment. Despite the increased number of POMC neurons in the ARC of the offspring of STZ-treated dams, this was not accompanied by changes in the POMC fiber density in the PVN (**Figure S1**, p>.05).

## 4 Discussion

This study was designed to further investigate the effects of glycemic dysregulation during pregnancy on the development and function of the POMC system as a mechanism promoting vulnerability to the development of metabolic disorders in the offspring. Our data show that STZ administration during pregnancy led to outcomes that were similar to those observed in women diagnosed with GDM, including glucose intolerance, increased risk for macrosomia, loss of pups at birth, as well as offspring vulnerability to develop metabolic alterations in adulthood, all confirming previous studies using a similar STZ model (29, 32, 33). Importantly, this study also shows that there are sex-specific effects of maternal STZ treatment in the offspring including decreased number of POMC neurons in the ARC on PD 19 of female but not male offspring. Furthermore, we also observed a higher number of POMC neurons in the ARC of adult male and female offspring born to STZ-treated dams, and, in females, this effect was exacerbated by exposure to HFD after weaning.

The hypothalamic melanocortin system has been established as a key node integrating hormonal and neuronal signals conveying information about peripheral energy state to regulate physiological and behavioral processes important for energy homeostasis. Critical to this system are cells that express POMC, a precursor peptide to α-melanocyte stimulating hormone (α-MSH), and one that increases energy expenditure while having a potent anorectic effect (34). Importantly, while lower POMC expression in the ARC is often viewed as a correlate for adult obesity (35, 36), several reports show that prolonged exposure to obesogenic diets or to early life obesogens lead to increased POMC expression in the ARC, arguing that overexpression of POMC in the ARC occurs as part of an allostatic process that is engaged to maintain homeostasis (37, 38). Therefore, the higher number of POMC neurons might be compensated in order to reduce food intake. Others suggest that overexpression of POMC is the result of increased cellular stress on POMC neurons that results in reduced cleavage of this pre-propeptide and ultimately increased POMC expression accompanied by lower levels of α-MSH (39, 40). In addition, changes in the expression endopeptidases that convert POMC into further neuropeptides, such as PC1/3, PC2, and furin, have also been implicated in the development of obesity (40) and might be altered following maternal hyperglycemia. In this sense, our data confirm previous data using a similar STZ model on mice and shows that POMC neurons in the ARC of female offspring may be particularly vulnerable to maternal hyperglycemia (28).

Changes in POMC expression may emerge during late gestation. As observed from our immunocytochemical data, the number of POMC neurons per section of the ARC was significantly lower in female fetuses harvested on PD 19 from STZ-treated dams, compared to that of females from control dams, showing that even a mild hyperglycemic environment *in utero* may induce changes in POMC neurons. It is uncertain if this reduction in POMC neurons' number is due to an overall cell reduction in the ARC, as previously described in other hypothalamic nuclei (27), a decrease in the generation of neurons, or simply a change in their phenotypic fate. The latter might be a potential mechanism given that there is a subset of POMC neurons that change into orexigenic neuropeptide Y (NPY)/agouti related peptide (AGRP) late in gestation *via* changes in the expression of homeobox and basic helix-loop-helix (bHLH) transcription factors (see (41) for review). Importantly, this would also suggest that a decrease in POMC staining through this mechanism would result in a concomitant increase in cells that produce NPY/AGRP, altering the balance between these two neuronal populations to bias the system towards a state that would promote higher caloric intake, weight gain, and adiposity, as observed in adult females from this study. Interestingly, in adulthood, there was an increase in POMC neurons in the offspring of STZ-treated dams, showing that postnatal development of the melanocortin system was also affected by maternal hyperglycemia. Despite the increased number of POMC neurons following maternal hyperglycemia and HFD intake in adulthood, there were no changes in POMC projections to the PVN, which might also explain why a higher number of POMC neurons was not sufficient to decrease food intake in those offspring. Overall, maternal hyperglycemia increased the vulnerability of females developing metabolic impairments, since they showed increased body weight gain, and this was associated with changes in gestational and adult POMC protein immunocytochemical expression in ARC.

Although STZ treatment mimics only the hyperglycemia seen in GDM, the similarities in outcomes observed in the current study highlight the relevance of this model as a preclinical model to study gestational diabetes. As it does not mimic the hyperinsulinemia of GDM, this experimental model is important to dissect which maternal and fetal outcomes are related to high glucose levels only. Like women diagnosed with GDM, STZ-treated rats showed mild hyperglycemia and altered glycemic regulation, as demonstrated by reduced glucose clearance and higher area under the curve following an OGTT. Interestingly, STZ treatment did not affect litter size, fetal, and placental weight in late pregnancy (PD 19), but it resulted in decreased litter size and increased offspring birth weight at birth. The loss of offspring in the last 48-72 hours of pregnancy, or shortly after birth, suggests that maternal hyperglycemia during pregnancy, like GDM, leads to an increased risk of late pregnancy complications that compromise fetus survival. Since dams were left with the litter throughout lactation, it was not possible to evaluate post-implantation loss in those animals, therefore we cannot determine whether pups died before birth or within the 24h between birth and litter culling. An increase in the size of the pups at birth, which was also described previously (32), is consistent with the macrosomia that is a frequent outcome in pregnancies complicated by GDM (5, 8, 9, 42), and one that leads to long-term negative metabolic consequences in the development of these children (43). Although fetal glucose and insulin levels were not evaluated on PD 19, fetal hyperinsulinemia has been reported in the offspring of STZ-treated dams in late pregnancy (33) and might contribute to the programming of macrosomia and obesity vulnerability later in life (8, 16). Overall, this supports the validity of this experimental preclinical model to study the mechanisms underlying the impact of GDM in humans.

Our data also reflect the inherent ability of homeostatic processes to adapt to challenges produced by the gestational and post gestational environment. For instance, while there were effects that clearly showed maternal impairments in glucose regulation in STZ-treated dams, and outcomes on the offspring at birth, long-term outcomes in offspring were not very different in pups from STZ-treated dams compared to pups from control dams. Although male pups from STZ-treated dams consumed more calories than control male pups while on a normal chow diet, there were no differences in their weight gain or adiposity. Chow-fed females showed very similar metabolic phenotypes regardless of whether their mother received STZ or vehicle injections. However, the offspring exposed to an HFD after weaning demonstrated vulnerability to the fat deposition that was sex-dependent, where females from STZ-treated dams put on more abdominal fat while males from these same dams put on less abdominal fat. This is in agreement with other models that lead to maternal hyperglycemia including studies demonstrating that female offspring are more susceptible to increased body weight gain and fat accumulation than their male littermates when they are born to HFD-fed dams (44). Additionally, female rats born to mothers fed with a high-fat/high-sugar diet during pregnancy perform less voluntary physical activity than males (45), which may explain differences in fat deposition, especially when exposed to obesogenic diets such as an HFD.

Despite inducing changes in fat deposition, maternal hyperglycemia did not affect offspring glucose tolerance in early adulthood, as only the HFD intake led to impaired glucose tolerance in both males and females. The absence of a maternal hyperglycemia effect may be due, in part, to increases in POMC expression in the ARC that were observed following maternal hyperglycemia and HFD exposure, which might increase insulin sensitivity and, thus, glucose tolerance (46). It is important to highlight that the offspring were evaluated at 60 days of age, which might be too early to detect metabolic impairments due to maternal hyperglycemia. Future studies should evaluate the development of metabolic disorders across life, especially in late adulthood.

This work suggests that maternal hyperglycemia produced by STZ treatment, in combination with early life exposure to an obesogenic diet leads to adult behavioral and metabolic alterations that correlate with increased expression of hypothalamic POMC to promote obesity. Furthermore, we show that female offspring of STZ-treated dams are more vulnerable to these effects. Ultimately, these data further demonstrate that maternal glycemic dysregulation can impact the development of hypothalamic circuits regulating energy state, having a stronger impact on female offspring. Importantly, these data also suggest that mitigation of these effects during pregnancy could prevent the long-lasting negative metabolic consequences on offspring.

## Data availability statement

The raw data supporting the conclusions of this article will be made available by the authors, without undue reservation.

## Ethics statement

The animal study was reviewed and approved by Carleton University Animal Care Committee.

## Author contributions

Conceptualization: MM, AK, AA; Formal analysis: MM, ZS, KA; Funding acquisition: AA; Investigation: MM, ZS, KA, LH; Supervision: AK, AA; Writing – Original draft preparation: MM, AA; Writing – Review & Editing: BW, AK. All authors contributed to manuscript revision, and read and approved the submitted version.

## Supplementary material

Supplementary Figure 1 POMC projections to the PVN in female and male offspring on PND 60. Maternal hyperglycemia and HFD intake did not change the density of POMC fibers in the PVN. Values expressed as mean ± standard error of the mean (Females: Control-chow N = 6; Control-HFD N = 4; STZ-Chow N = 6; STZ-HFD N = 5. Males: Control-chow N = 5; Control-HFD N = 5; STZ-Chow N = 6; STZ-HFD N = 3). The images represent POMC staining in the PVN in all experimental groups. The scale in the first image (100 µm) is valid for all the images.
