# Supplementary figures and images for: Maternal glucose intolerance during pregnancy affects offspring POMC expression and results in adult metabolic alterations in a sex-dependent manner
Source: Front Endocrinol (Lausanne). 2023 Jun 15;14:1189207. doi: 10.3389/fendo.2023.1189207 (PMC10311085; doi:10.3389/fendo.2023.1189207)

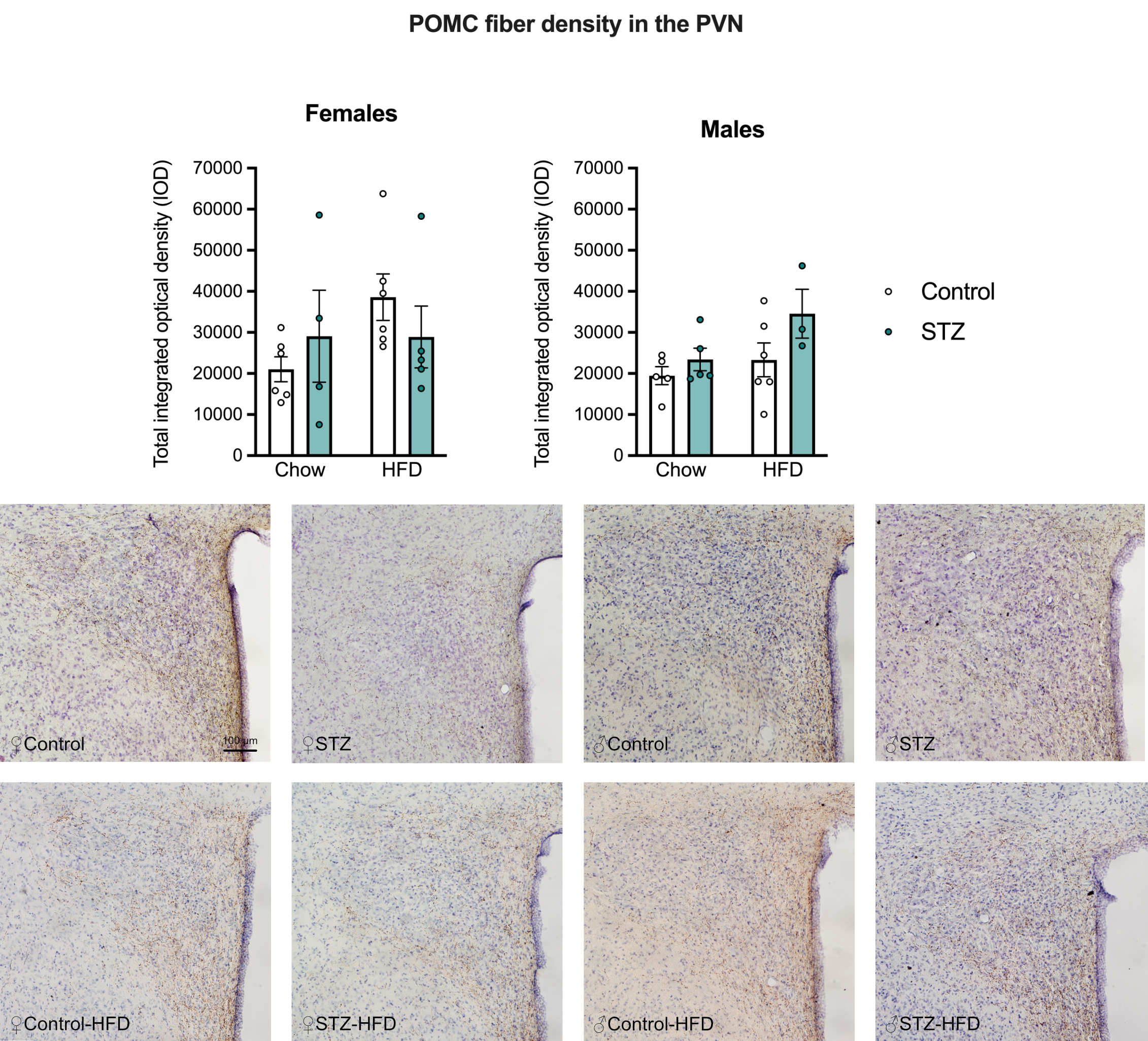

Supplement: Supplementary Figure 1 — POMC projections to the PVN in female and male offspring on PND 60. Maternal hyperglycemia and HFD intake did not change the density of POMC fibers in the PVN. Values expressed as mean ± standard error of the mean (Females: Control-chow N = 6; Control-HFD N = 4; STZ-Chow N = 6; STZ-HFD N = 5. Males: Control-chow N = 5; Control-HFD N = 5; STZ-Chow N = 6; STZ-HFD N = 3). The images represent POMC staining in the PVN in all experimental groups. The scale in the first image (100 µm) is valid for all the images. [file Image_1.tiff]
